# *Pseudomonas aeruginosa* countrywide outbreak in hospitals linked to pre-moistened non-sterile washcloths, Norway, October 2021 to April 2022

**DOI:** 10.2807/1560-7917.ES.2022.27.18.2200312

**Published:** 2022-05-05

**Authors:** Kirsten Gravningen, Oliver Kacelnik, Egil Lingaas, Torunn Pedersen, Bjørn G Iversen, the Pseudomonas outbreak group, The Pseudomonas outbreak group, Anne M Asfeldt, Anita Blomfeldt, Bente C Borgen, Petter Elstrøm, Gro Grimnes, Nils O Hermansen, André Ingebretsen, Jessin Janice, Silje B Jørgensen, Kristin S Kilhus, Nicola I. Kols, Julie A Korpås, Siv A Kvaal, Pia Littauer, Birgitte Lyrån, Iren H Løhr, Marie T Noer, Anette Skjærvik, Dag H Skutlaberg, Arnfinn Sundsfjord, Mari N Utheim, Liz E Ødeskaug

**Affiliations:** 1 Norwegian Institute of Public Health, Oslo, Norway; 2 Department of Infection Prevention, Oslo University Hospital, Oslo, Norway; 3 Norwegian National Advisory Unit on Detection of Antimicrobial Resistance, University Hospital of North Norway, Tromsø, Norway; 4 The members of the group are listed under Collaborators

**Keywords:** Pseudomonas aeruginosa, hospital outbreak, non-sterile product, nosocomial infection, healthcare-associated infection, hospital environment, whole-genome sequencing, Gram-negative bacteria

## Abstract

In November 2021, a clonal outbreak of *Pseudomonas aeruginosa* of novel sequence type ST3875 was detected in three patients who died of bloodstream infections in one hospital. By 25 April 2022, the outbreak included 339 cases from 38 hospitals across Norway. Initial hospital reports indicate *Pseudomonas* infection as the main contributing cause in seven deaths. In March 2022, the outbreak strain was identified in non-sterile pre-moistened disposable washcloths, used to clean patients, from three lots from the same international manufacturer.

On 19 November 2021, the Norwegian Institute of Public Health (NIPH) was notified by the University Hospital of North-Norway, Tromsø that three patients hospitalised with severe COVID-19 in the intensive care unit had died of bloodstream infections (BSIs) with indistinguishable *Pseudomonas aeruginosa* strains, only a few days apart. Whole genome sequencing (WGS) revealed a novel sequence type. Further cases were identified in several other hospitals thereafter; here we describe the outbreak and preliminary findings.

## Timeline of the outbreak

Cases were rapidly detected in hospitals in all four health regions in Norway, strongly indicating a common source of infection [1-5]. *Pseudomonas aeruginosa* is an opportunistic pathogen associated with nosocomial infections that thrives in humid environments [6].

On 18 March 2022, Oslo University Hospital detected *P. aeruginosa* in disposable pre-moistened washcloths after systematic testing of several hundred different products that were used in hospital wards with identified cases. Particular attention was paid to moist products that had not been terminally sterilised by the manufacturer such as liquid soaps, hand creams, toothpaste and lubricating gels for endoscopic procedures. Samples of the washcloths were cultivated by standard microbiological procedures [7]. On the same day, the hospital notified the NIPH and Norwegian Hospital Procurement Trust about the product type and lot number. The latter immediately alerted all hospitals and the Norwegian distributor that the hospitals should stop using these products. On 19 March, the *P. aeruginosa* isolates were confirmed as the outbreak strain ST3875 by clone-specific PCR and later through WGS. On 21 March, the detection of the probable source of infection was published on the NIPH website [8]. By 25 April, ST3875 had been detected by seven different hospital laboratories in 149 of the 577 washcloths tested from four lots produced on multiple dates [9].

The manufacturer notified their customers in Norway on 21 March. The Norwegian Food Safety Authority (NFSA) banned the contaminated lots from the Norwegian market on 23 and 30 March. The responsible person (RP) for the product in EU/European Economic Area (EEA) was contacted by the NFSA – through the Competent Authority in the country where the RP was based. On 4 April, NFSA notified the EU countries through the EU rapid alert system for unsafe consumer products (RAPEX).

The RP for a cosmetic product throughout the EU/EAA markets is a legal representative who is obliged to ensure that the product is safe and meets regulatory requirements and shall take immediate corrective action (recall or withdrawal) if necessary [10].

The manufacturer’s initial laboratory test reports of the relevant product lots conveyed via the RP to NFSA were negative for *P. aeruginosa*. However, retesting of retained samples confirmed the presence of *P. aeruginosa* in the same lots. As part of the RP internal control, NFSA was able to identify additional product lots that had been incorrectly released by the manufacturer after *P. aeruginosa* was detected and also found discrepancies in other test reports. On 9 April, the NFSA released a warning in Norway against use of all products from this manufacturer.

On 7 April, the RP visited the manufacturer. On 14 April, the findings led to a voluntary recall of all lots of a total of 14 different products from one facility, issued to customers in the EU/EEA. The recall was done in order to ensure the highest level of consumer safety. This decision was supported by the Competent Authority which also notified EU countries through RAPEX.

**Figure 1:**
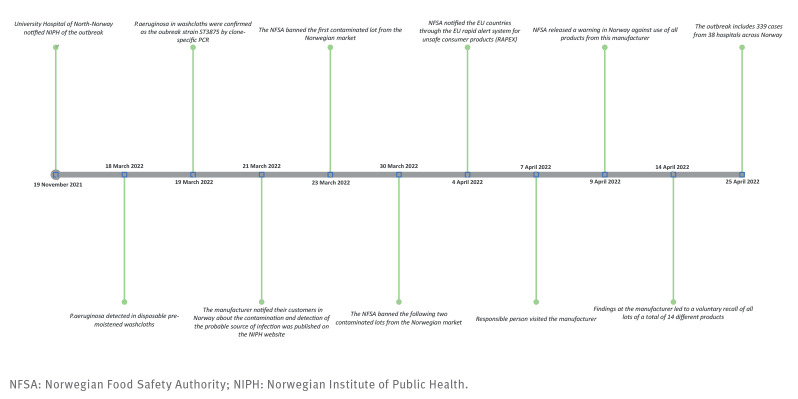
Timeline of investigation into *Pseudomonas aeruginosa* cases in hospitals, Norway, October 2021–April 2022 (n = 339)

NFSA: Norwegian Food Safety Authority; NIPH: Norwegian Institute of Public Health.

The complete timeline of events is presented in Figure 1.

## Setting

Cases included in the ongoing outbreak are from 15 October 2021 to 25 April 2022.

## Outbreak investigation

A national outbreak group coordinated by the NIPH, including professionals from all health regions, was established on 20 January 2022. A confirmed case was defined as a hospitalised patient who was infected or colonised with *P. aeruginosa* ST3875.

The national outbreak group recommended that all hospitals should conduct targeted screening of patients in intensive care units and genotyping of *P. aeruginosa* strains detected in diagnostic and clinical samples. All samples were analysed in local hospital laboratories. In addition, most laboratories retrospectively genotyped *P. aeruginosa* in stored blood culture samples starting 1 January 2021. Initially, *P. aeruginosa* isolates were characterised using WGS or amplified fragment length polymorphism (AFLP) assay [11]. An in-house clone-specific real-time PCR was developed and validated by one hospital laboratory by week 8, 2022, and the PCR specifications were rapidly shared with other laboratories.

NIPH initiated two parallel lines of investigation. For the first line, each case was notified by the hospitals to the NIPH together with a comprehensive record of risk factors, exposures and clinical outcomes. The second line involved compiling a list of products purchased such as mouth swabs, hygiene products, urinary catheters, infusion sets and lidocaine gel during the outbreak period on the wards where the cases were cared for. However, the latter analyses revealed no indication of products that could be a possible source of the outbreak.

## Demographic and clinical information

In total, 339 cases from 38 hospitals with a median age of 70 years (interquartile range: 59-78 plus one child) have been identified. The cases included in the outbreak register are described in the Table.

**Table:** Characteristics of cases, outbreak of *Pseudomonas aeruginosa* ST3875 infections in 38 hospitals across all four health regions in Norway, October 2021–April 2022 (n = 339)

Case characteristics	n	%
**Sex**		
Women	123	36.3
Men	216	63.7
**Age (years)**		
Median (IQR)	70 (59–78)	
Mean (SD)	67.5 (15.8)	
**Health region** ^a^		
Northern	30	8.8
Central	47	13.9
South-eastern	241	71.1
Western	21	6.2
**Hospital ward** ^b^		
Intensive care unit	129	38.1
General ward	164	48.4
Emergency ward	10	3.0
Other	36	10.6
**Colonisation vs infection**		
Colonisation only	65	19.2
Mild to moderate infection	190	56.0
Severe infection^c^	83	24.5
Missing information	1	0.3
***Pseudomonas* as cause of death** ^d^ **(n=53)**		
Strongly contributing	7	13.2
Possibly contributing	22	41.5
Not contributing	20	37.7
Unknown	4	7.6

IQR: interquartile range; SD: standard deviation.

^a^ The South-eastern health region is the largest region and provides specialist healthcare to 58% of the population in Norway.

^b^ Type of ward the patient was staying on when they had the first positive test for *P. aeruginosa* ST3875.

^c^ The severity of infection was assessed by a physician.

^d^ The role of *Pseudomonas* in the cause of death was assessed by a physician (the time between death and this assessment varied by patient). Final information is not available for all patients.

The presented information is based on data reported by hospital or infection control staff in each hospital.

Disease severity assessment and the cause of death review were done at the local level by a physician. Fifty-three deaths (16%) have so far been reported and for these, *Pseudomonas* infection was considered the main contributing cause of death in seven cases.

**Figure 2:**
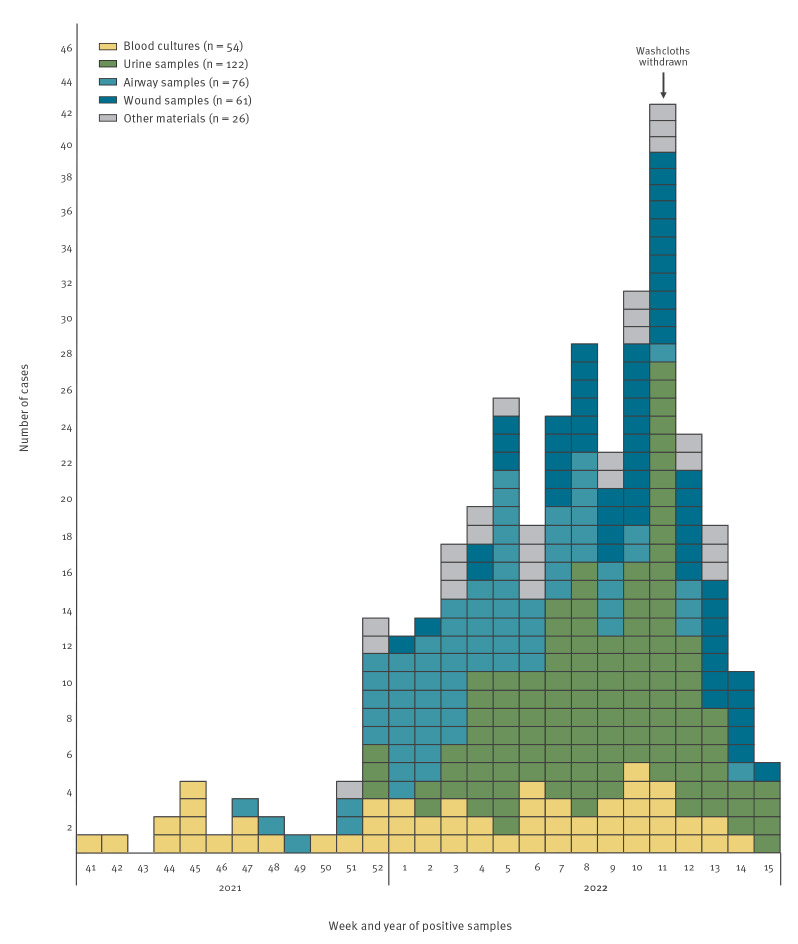
Distribution of *Pseudomonas aeruginosa* ST3875 cases by test date (week) for the first positive sample, by case and sample type, 38 hospitals, Norway, October 2021–April 2022 (n = 339)

We included the first positive sample for each patient (n = 339). These samples were taken from diverse body sites indicating multiple routes of entry, including 16% blood cultures, 36% urine samples, 22% airway samples, 18% wound samples and 8% from other locations (Figure 2). 

One sample per patient is included in the figure. For patients who had a positive blood culture sample, this sample is included in the figure, even if the patient had an earlier positive sample from another body site (n = 4). Eleven blood cultures between weeks 41 and 51 were analysed retrospectively as part of the outbreak investigation.

After use of the product was stopped between 18 and 20 March (week 11), we observed a rapid decline in cases over the next 4 weeks until 25 April. However, there may be a reporting delay. Screening is ongoing and the hospitals will continue to report new cases to NIPH.

### Genomic epidemiology

**Figure 3:**
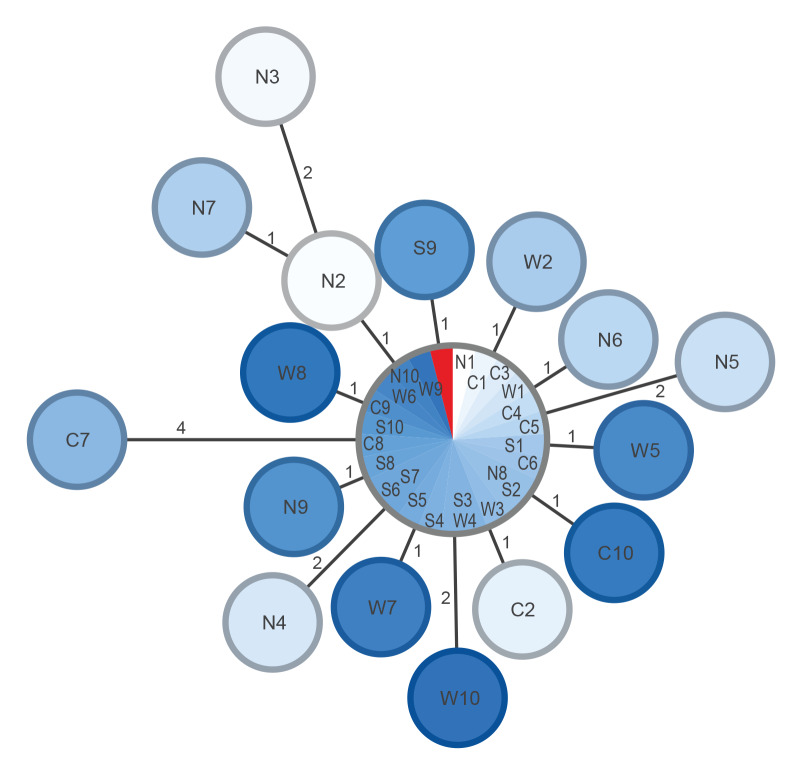
Minimum spanning tree of selected clinical *Pseudomonas aeruginosa* ST3875 strains (n=40) from the four health regions and one selected washcloth isolate, Norway, October 2021–April 2022

Genomic analyses were performed on quality-assessed Illumina-based assemblies by core genome phylogeny (Ridom SeqSphere+ version 8.3) based on 3,867 target genes [12]. Figure 3 illustrates the clonal relatedness of selected clinical strains from the four health regions and one selected washcloth isolate. The analysis showed an outbreak cluster with 0–4 allelic differences and most of the strains, including the one from the washcloth, present in the same node.

Included health regions: N = Northern, C = Central, S = South-eastern, W = Western. The colour gradient of nodes reflects the dates of strain isolation spanning 15 October 2021 (light blue) to 17 March 2022 (dark blue), with 10 strains from each region numbered chronologically. The washcloth isolate is shown in red.

## Discussion

Our preliminary investigation of the clonal *P. aeruginosa* ST3875 hospital outbreak in Norway revealed that contaminated non-sterile washcloths used for intensive care and other vulnerable patients were the source of the outbreak and have caused a number of infections, many of them severe, and contributed to at least seven deaths. Even though most cases were colonised or had mild urinary tract and wound infections there have been several severe infections including ventilator-associated pneumonia and BSIs among reported cases.

The clinical strains of *P. aeruginosa* ST3875 displayed a wild-type antimicrobial susceptibility profile [13]. However, resistance towards anti-pseudomonas drugs was discovered after antimicrobial treatment of single patients (data not shown)*.* Thus, had it not been for the close proximity of the three initial BSI cases (time and place), the outbreak may have gone undetected for a longer period. The pre-moistened washcloth was distributed and used all over Norway, including in primary healthcare. According to the manufacturer’s website, this product is used in healthcare worldwide.

The washcloth is classified as a non-sterile cosmetic product and does not have to meet the standards for medical devices and medicines. However, every cosmetic manufacturer is responsible for the microbiological safety of its products to ensure that they have been produced under hygienic conditions. *P. aeruginosa* is one of four species (in addition to *Staphylococcus aureus*, *Escherichia coli* and *Candida albicans*) for which cosmetic products should be tested and contamination excluded, according to the Commission Implementing Decision on guidelines on Annex I to Regulation (EC) No 1223/2009 on cosmetic products [10,14,15].

The use of this product is widespread but often not documented in patient records and thus difficult to investigate in traditional epidemiological studies. According to the hospital line lists, 40% of cases had been exposed to disposable washcloths, 6% had not, and 54% reported 'unknown' or had missing information. However, one hospital systematically tested several hundred suspected products in their laboratory, thereby detecting the infection source. The manufacturer’s control routines did not detect *P. aeruginosa*.

According to the manufacturer, three of four lots where contamination has been detected have only been distributed to Norway. However, without knowing the system of production lines, quantity produced per lot and assignment of lot numbers, it remains difficult to identify exactly the contaminated lots and to outline an appropriate future test strategy for this product in Norway. Norway plans to evaluate the routines for the use of this kind of non-sterile product in healthcare settings, especially among intensive care patients and other vulnerable patients [4].

## Conclusion

The products have been sold and used in many countries and therefore this outbreak may well be ongoing and undetected in several EU states and beyond. Consequently, it is prudent to advise further investigations of *P. aeruginosa* ST3875 infections, and when identified, investigate if they are linked to the same product.

## The Pseudomonas outbreak group

Anne M Asfeldt (Department of Microbiology and Infection Control, University Hospital of North Norway, Tromsø, Norway, Department of Community Medicine, UiT – The Arctic University of Norway, Tromsø, Norway), Anita Blomfeldt (Department of Microbiology and Infection Control, Akershus University Hospital, Lørenskog, Norway, Department of Clinical Molecular Biology (EpiGen), Akershus University Hospital and University of Oslo, Lørenskog, Norway), Bente C Borgen (Department of Infection Prevention, Oslo University Hospital, Oslo, Norway), Petter Elstrøm (Norwegian Institute of Public Health, Oslo, Norway), Gro Grimnes (Division of Internal Medicine, University Hospital of North Norway, Tromsø, Norway, Department of Clinical Medicine, UiT-The Arctic University of Norway, Tromsø, Norway), Nils O Hermansen (Department of Microbiology, Oslo University Hospital, Oslo, Norway), André Ingebretsen (Department of Infection Prevention, Oslo University Hospital, Oslo, Norway, Department of Microbiology, Oslo University Hospital, Oslo, Norway), Jessin Janice (Norwegian National Advisory Unit on Detection of Antimicrobial Resistance, University Hospital of North Norway, Tromsø, Norway), Silje B Jørgensen (Norwegian Institute of Public Health, Oslo, Norway, Department of Microbiology and Infection Control, Akershus University Hospital, Lørenskog, Norway), Kristin S Kilhus (Western Norway Advisory Unit on IPC, Department of Research and Development, Haukeland University Hospital, Bergen, Norway), Nicola I Kols (Regional Centre for Infection Prevention and Control, Central Norway Regional Health authority, St. Olavs Hospital, Trondheim University Hospital, Trondheim, Norway), Julie A Korpås (Norwegian Institute of Public Health, Oslo, Norway), Siv A Kvaal (Regional Centre for Infection Prevention and Control, Central Norway Regional Health authority, St. Olavs Hospital, Trondheim University Hospital, Trondheim, Norway), Pia Littauer (Department of Microbiology and Infection Control, University Hospital of North Norway, Tromsø, Norway), Birgitte Lyrån (The Norwegian Food Safety Authority, Oslo, Norway), Iren H Løhr (Department of Medical Microbiology, Stavanger University Hospital, Stavanger, Norway), Marie T Noer (Department of Microbiology, Oslo University Hospital, Oslo, Norway), Anette Skjærvik (Department of Medical Microbiology, St. Olavs Hospital, Trondheim University Hospital, Trondheim, Norway), Dag H Skutlaberg (Department of Microbiology, Haukeland University Hospital, Bergen, Norway), Arnfinn Sundsfjord (Norwegian National Advisory Unit on Detection of Antimicrobial Resistance, University Hospital of North Norway, Tromsø, Norway, Department of Medical Biology, Faculty of Health Sciences, UiT – The Arctic University of Norway, Tromsø, Norway), Mari N Utheim (Norwegian Institute of Public Health, Oslo, Norway), Liz E Ødeskaug (Norwegian Institute of Public Health, Oslo, Norway).
